# Role of auxin (IAA) in the regulation of slow vacuolar (SV) channels and the volume of red beet taproot vacuoles

**DOI:** 10.1186/s12870-018-1321-6

**Published:** 2018-06-04

**Authors:** Zbigniew Burdach, Agnieszka Siemieniuk, Zenon Trela, Renata Kurtyka, Waldemar Karcz

**Affiliations:** 1 Department of Plant Physiology, Faculty of Biology and Environmental Protection, University of Silesia, Jagiellońska 28, 40-032 Katowice, Poland; 2 Department of Physics and Biophysics, Wrocław University of Environmental and Life Sciences, Norwida 25, 50-375 Wrocław, Poland

**Keywords:** *Beta vulgaris* L., IAA (indole-3-acetic acid), SV channels, Vacuole, Vacuolar volume

## Abstract

**Background:**

Auxin (IAA) is a central player in plant cell growth. In contrast to the well-established function of the plasma membrane in plant cell expansion, little is known about the role of the vacuolar membrane (tonoplast) in this process.

**Results:**

It was found that under symmetrical 100 mM K^+^ and 100 μM cytoplasmic Ca^2+^ the macroscopic currents showed a typical slow activation and a strong outward rectification of the steady-state currents. The addition of IAA at a final concentration of 1 μM to the bath medium stimulated the SV currents, whereas at 0.1 and 10 μM slight inhibition of SV currents was observed. The time constant, τ, decreased in the presence of this hormone. When single channels were analyzed, an increase in their activity was recorded with IAA compared to the control. The single-channel recordings that were obtained in the presence of IAA showed that auxin increased the amplitude of the single-channel currents. Interestingly, the addition of IAA to the bath medium with the same composition as the one that was used in the patch-clamp experiments showed that auxin decreased the volume of the vacuoles.

**Conclusions:**

It is suggested that the SV channels and the volume of red beet taproot vacuoles are modulated by auxin (IAA).

## Background

Auxins, particularly indole-3-acetic acid (IAA), play an essential role in the regulation of plant cell extension. According to the so-called “acid growth theory”, auxin activates the PM H^+^-ATPase, which acidifies the apoplast and causes the activation of the enzymes that are involved in cell wall loosening (for a review see [1]). It is also well established, at least in maize coleoptile cells, that auxin-induced growth involves K^+^ uptake through voltage-dependent, inwardly rectifying K^+^ channels (ZMK1, *Zea mays* K^+^ channel 1), the activity of which contributes to water uptake and consequently to cell expansion [2, 3]. It has been shown that apart from the posttranslational, auxin-dependent up-regulation of the K^+^ uptake channels, auxin also regulates the expression of the maize K^+^ uptake channel gene *ZMK1* [2]. ZMK1 channels are activated by a hyperpolarizing membrane potential and by extracellular apoplastic protons.

Significantly less is known about the role of the vacuolar membrane, the tonoplast, in the auxin-mediated growth of plant cells. Plant cells contain a large central vacuole that occupies up to 95% of the total cell volume in many mature plant cells. Plant cell expansion is driven by a combination of the osmotic uptake of water into the vacuoles and altered cell wall extensibility. To maintain the turgor pressure of expanding cells, solutes must be transported into the vacuole to maintain its osmolarity. Vacuoles are very dynamic organelles, whose morphology changes during plant growth and development [4, 5]. It has been shown that auxin (IAA) and its metabolites are present in plant vacuoles and that auxin transport across the tonoplast plays essential roles in maintaining auxin homeostasis [6]. It is also well known that auxin stimulates or inhibits the growth of plant cells depending on its concentration as well as the cell type [7]. Recently, it has been shown that auxin altered the appearance of the vacuoles in the root epidermal cells of *Arabidopsis thaliana* so that they became smaller [8]. At the same time, as these authors showed, auxin also inhibited the growth of the root epidermal cells. This finding was used by Dünser and Kleine-Vehn [9] to propose the “acid growth balloon theory” according to which plant growth is the interplay between the intracellular space-filling “vacuolar balloon” and the required extracellular cell wall acidification/loosening.

Taking into account that plant vacuoles are highly dynamic organelles and are essential for growth and development, we performed experiments in which the effect of auxin (IAA) on the slow vacuolar (SV) channels and the volume of red beet taproot vacuoles were studied. In the plant vacuoles, slow vacuolar (SV) channels are Ca^2+^-permeable cation channels that are coregulated by voltage and Ca^2+^. These SV channels are ubiquitous and abundant in the vacuolar membrane of terrestrial plants. The SV channel from *Arabidopsis*, TPC1, is encoded by the single-copy gene *AtTPC*1 [10]. Structurally, TPC1 represents a dimer of two *Shaker*-like monomers that are linked via a cytoplasmic loop that contains two EF hand motifs ([11, 12] for a review see [13]). The activity of voltage-dependent TPC1 channels can be regulated by both cytosolic and vacuolar Ca^2+^. Cytosolic Ca^2+^ promotes channels opening [12, 14], whereas luminal Ca^2+^ prevents their opening [15]. Three decades after the discovery of the SV channels by Hedrich et al. [16] and Hedrich and Neher [14], two groups of researchers revealed the essential structural and functional properties of TPC1/SV channels in *Arabidopsis thaliana* based on their crystal structure [17, 18]. Soon after, Jaślan et al. [19] published a paper in which the structural determinants of the voltage- and calcium-dependent channel gating of AtTPC1 were described. For their analysis, these authors built a three-dimensional homology model of AtTPC1 that was based on the crystal structure of the bacterial voltage-gated Na^+^ channel Na_v_Ab. To the best of our knowledge, no research has been reported on the effects of IAA on the TPC1/SV channels in plant cells. We hypothesize that SV channels representing the major cations conductance are involved in auxin-induced volume changes of the vacuoles.

## Results

### Effect of IAA on the volume of red beet taproot vacuoles

**Fig. 1:**
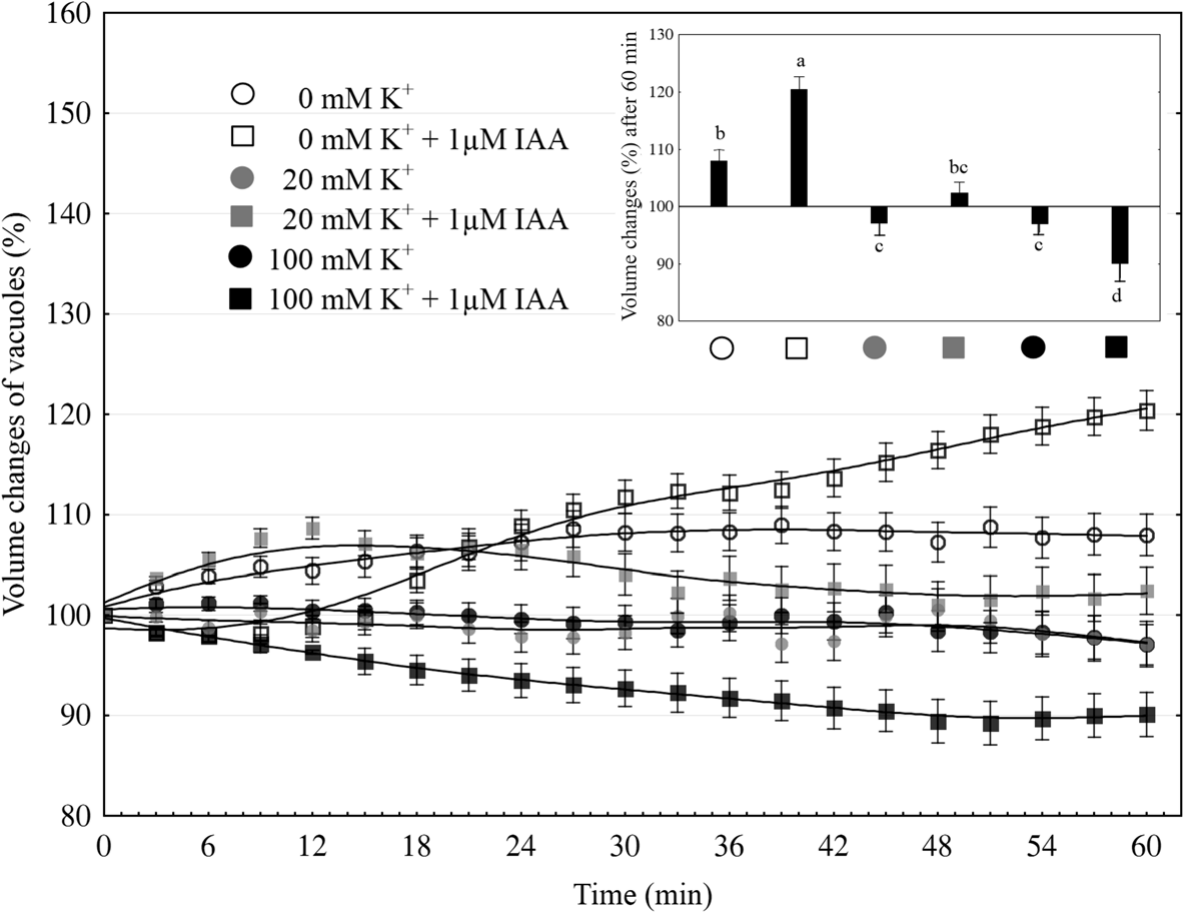
Effect of 1 μM indole-3-acetic acid (IAA) on the volume changes of vacuoles. The vacuoles had been incubated in the presence of K^+^ at 0, 20 and 100 mM. IAA was added to the incubation medium at time 0 min. The data points are the means (± SE) from nine independent experiments. The volume of individual vacuoles was calculated from the diameter of the individual vacuoles in a photographic image. The diameter of the vacuoles was measured at the indicated times and converted to a percentage of the initial value (fixed as 100%). The inset on the right shows the volume of vacuoles after one hour of the experiment. Bars indicate means ± SEs. Means followed by the same letter are not significantly different from each other (LSD test *P* < 0.05)

Red beet vacuoles were mechanically isolated directly onto glass slides by rinsing the surface of fresh tissue slices with a medium containing various K^+^ concentrations (0, 20 and 100 mM). As Fig. 1 indicates, the volume of the vacuoles that were incubated in the bath medium without K^+^ and with 1 μM IAA increased after 60 min up to 8% of their initial value (at 0 min). In the presence of IAA, the volume of the vacuoles that had been incubated in bath medium without K^+^ increased by 20%. When the vacuoles were incubated in the presence of 20 or 100 mM K^+^, their volume was about 3% lower compared to the first value. The addition of IAA to the bath solution with 20 mM K^+^ slightly increased (by 3% over 60 min) the volume of the vacuoles, while its addition to the medium with 100 mM K^+^ decreased (by 10% over 60 min) their volume. Interestingly, in the presence of both IAA and 100 mM K^+^, a decrease in the volume of the vacuoles was observed from the beginning of the experiment. The data obtained in this section clearly showed that the effect of IAA on the volume of red beet vacuoles depends on the potassium concentration.

### Electrophysiological experiments

**Fig. 2:**
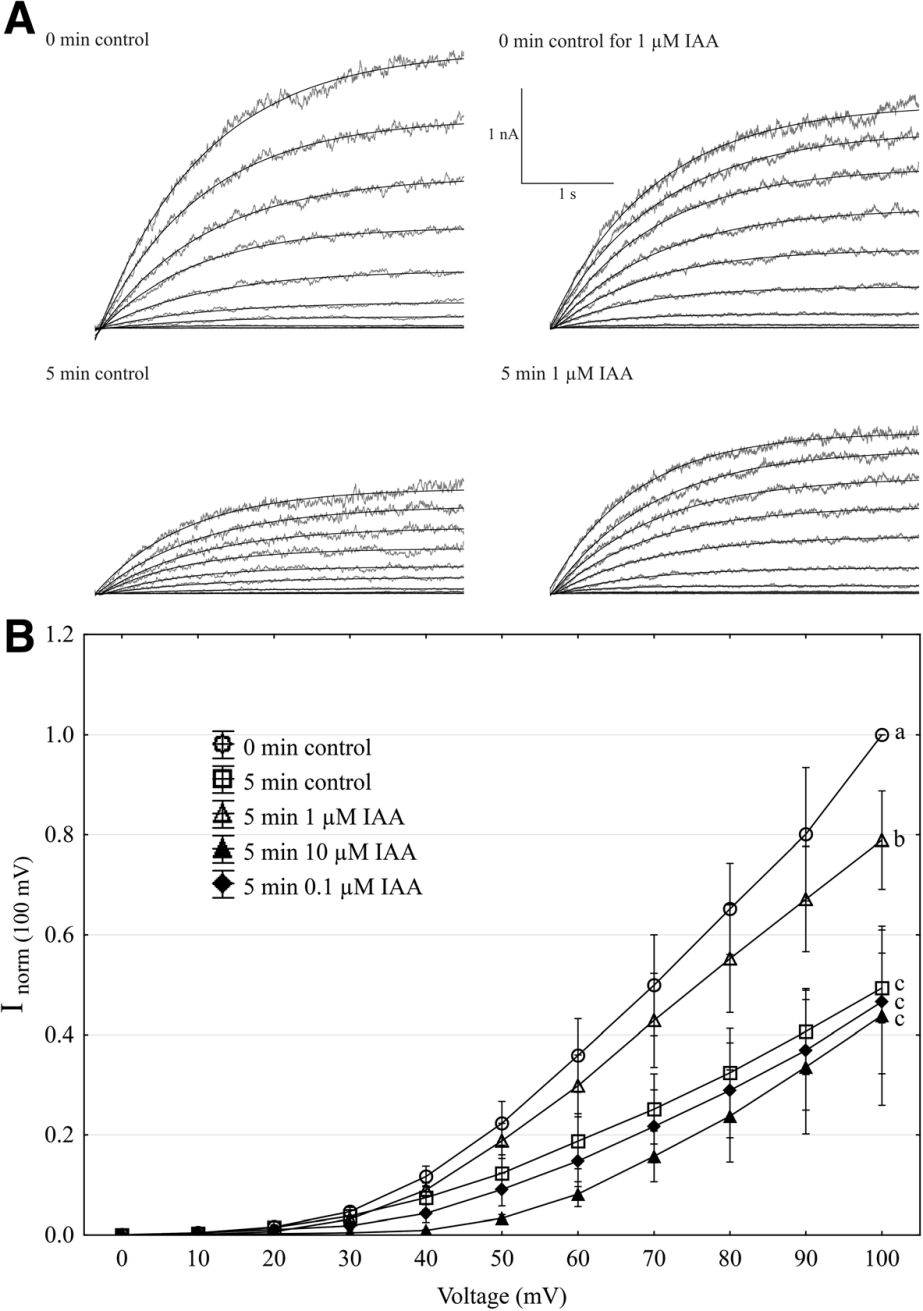
Effect of cytosolic IAA on the slow vacuolar (SV) channels in red beet taproot vacuoles. **a** An example of an SV current recording for a single vacuole in the control bath (control at 0 time, recorded immediately after the establishment of the whole-vacuole configuration as well as 5 min later) and in the presence of IAA at 1 μM (auxin was added to the bath immediately after the current was recorded in the control at 0 time; however, the current in the presence of IAA was recorded 5 min after the control at 0 time). SV currents elicited by a series of voltage steps ranging from − 100 to + 100 mV in 10 mV steps; holding potential 0 mV. **b** Steady-state currents (normalized to the current amplitude at + 100 mV under control at 0 min) were determined in the control medium (control at 0 and 5 min) and in the presence of 0.1, 1 and 10 μM IAA. The current traces were fitted with the exponential function: *i*(*t*) = *a* + *b* (1-exp(−*t/τ*)), where *a* - current at *t* = 0, *b* - current at saturation (plateau), *t* - time and *τ* - time constant. The steady state is the difference between current at saturation (plateau) and current at time “0” (leak). Data points are the means (± SE) from at least seven experiments performed with different vacuoles. The significance of the results was analyzed for voltage + 100 mV using the post hoc least significant difference (LSD) test. Means followed by the same letter are not significantly different from each other (LSD test *P* < 0.05)

**Fig. 3:**
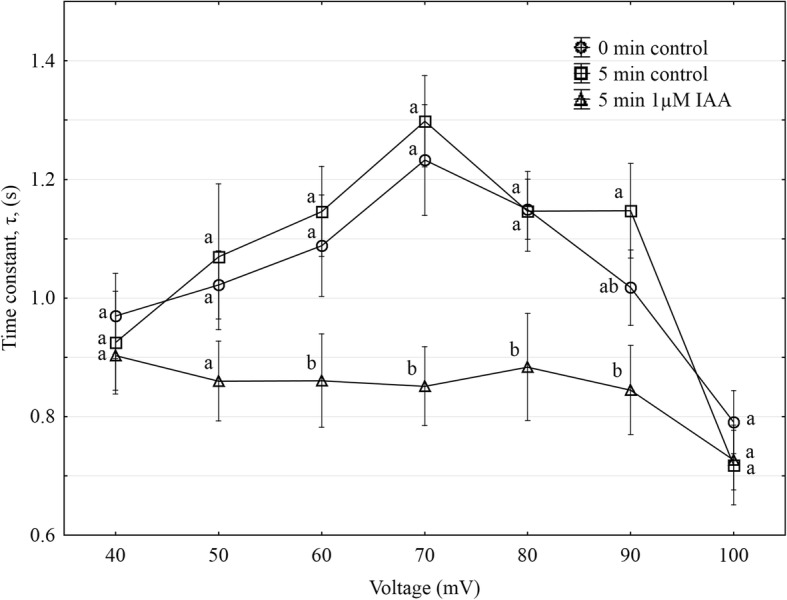
Effect of IAA at 1 μM on activation time, τ, as a function of voltage. Data points are the means (± SE) from at least seven experiments performed with different vacuoles. The significance of the results was analyzed for every voltage (+ 40 mV to + 100 mV) using the post hoc least significant difference (LSD) test. Means followed by the same letter are not significantly different from each other (LSD test *P* < 0.05)

**Fig. 4:**
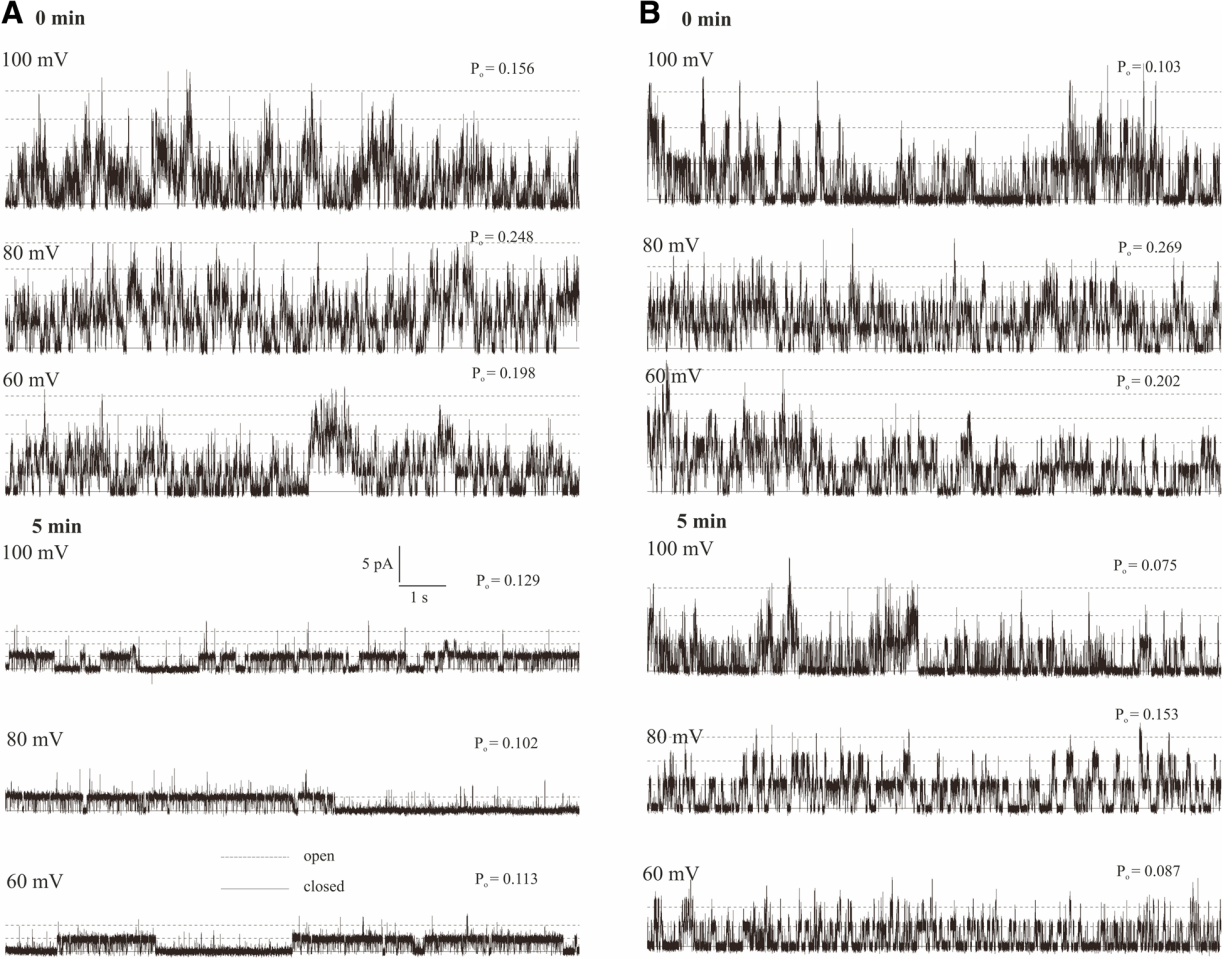
An example of the microscopic SV current traces at selected voltages. Single channel openings in the control bath (**a**) (control at 0 time and 5 min later, see explanation in Fig. 2) and in the presence of IAA at 1 μM (**b**) (the current in the presence of IAA was recorded 5 min after the control at 0 time) are shown. Single channel fluctuations were recorded at + 60, + 80 and + 100 mV. The solid line indicates closed state of the channels, the dashed line – open state. The values of open probabilities for single traces are presented as P_o_

**Fig. 5:**
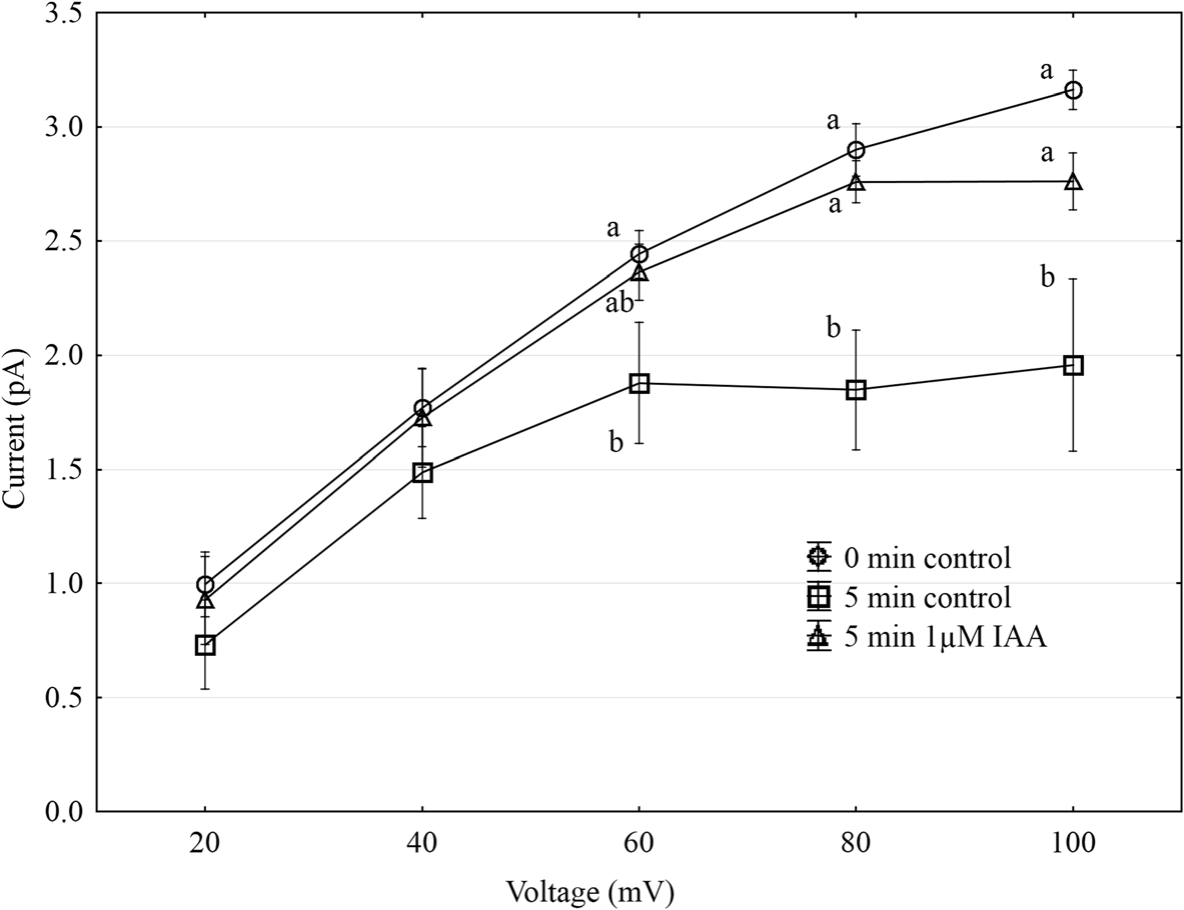
Current-voltage relationships for the microscopic SV currents. The currents were recorded in the control bath (control at 0 time and 5 min later) and in the presence of IAA at 1 μM. Points represent the means (± SE, *n* = 8) for the events of “open 1” that were recorded at selected voltages. The significance of the results was analyzed for every voltage (+ 60 mV to + 100 mV) using the post hoc least significant difference (LSD) test. Means followed by the same letter are not significantly different from each other (LSD test *P* < 0.05)

**Fig. 6:**
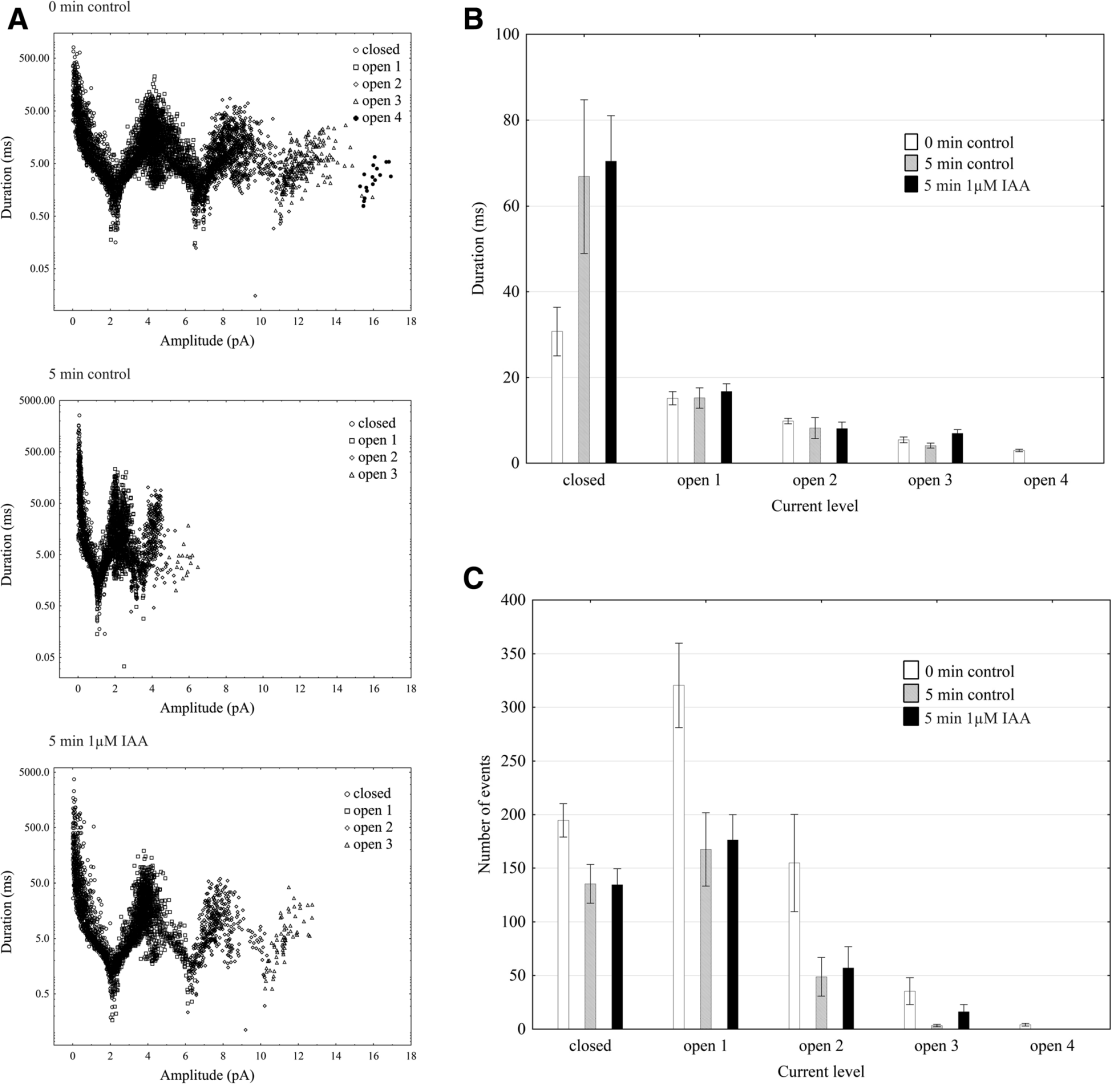
Distribution of the times of different current state events. The events (closed, open 1, 2, 3 and 4) were presented as a function of the amplitude of current events (i.e. its average value during an event) in the control (control at 0 min and 5 min later) and in the presence of IAA at 1 μM. All events were collected from eight current traces (each of 12 s duration) that were obtained at a voltage of + 100 mV (**a**). The average values of the times of the current events versus the current level (± SE, n = 8) (**b**). The average values of the number of events versus the current level (± SE, n = 8) (**c**). FitMaster software was used to analyze the opening events

**Fig. 7:**
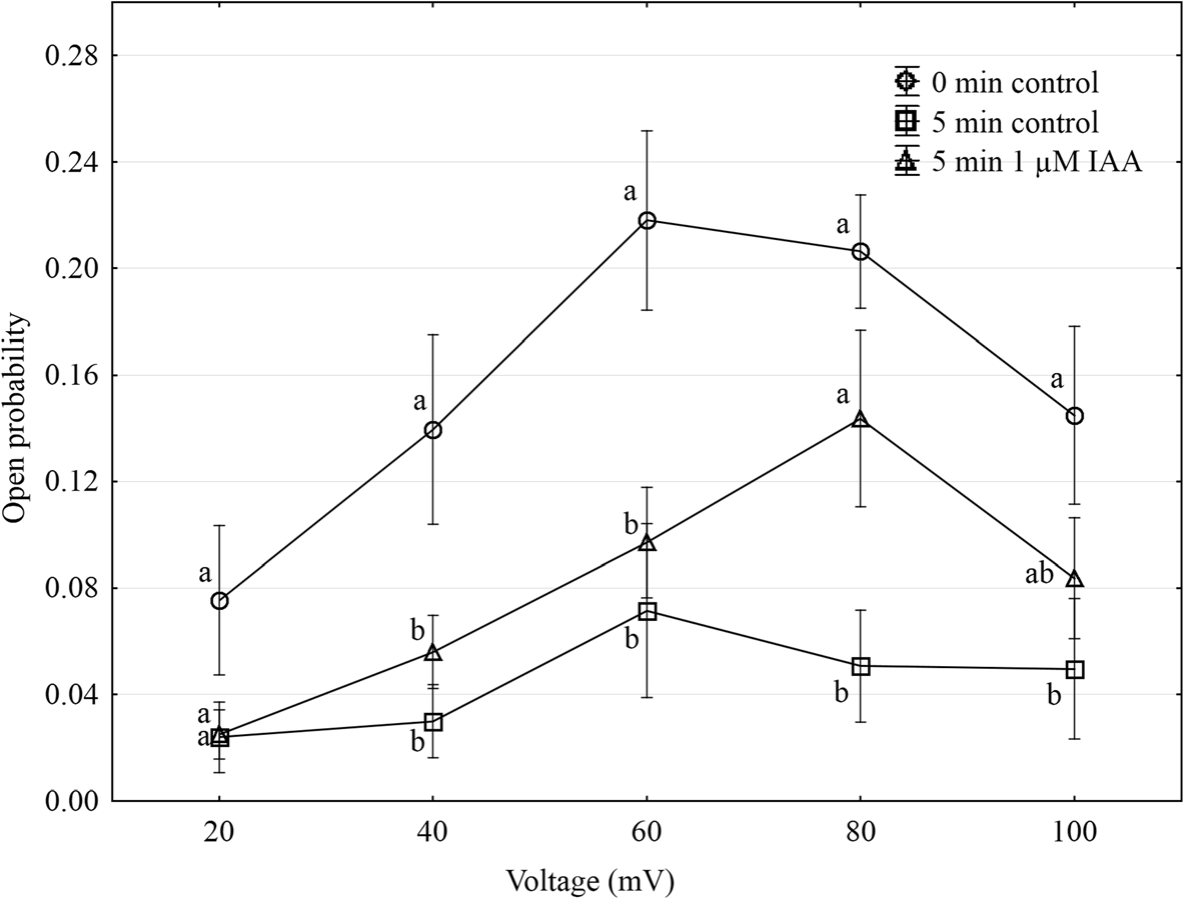
Open probability of the slow vacuolar (SV) channels as a function of voltage. The open probability was calculated (using FitMaster software) as the sum of the channel open time in the current traces that were normalized to the total time of the traces and divided by the number of active channels in the patch. Data points are the means (± SE) from eight independent experiments. The significance of the results was analyzed for every voltage using the post hoc least significant difference (LSD) test. The significance of the results was analyzed for every voltage (+ 20 mV to + 100 mV) using the post hoc least significant difference (LSD) test. Means followed by the same letter are not significantly different from each other (LSD test *P* < 0.05)

Using the patch-clamp technique, we examined the effect of IAA on the slow vacuolar (SV) channel activity in red beet (*Beta vulgaris* L.) taproot vacuoles. Both macroscopic currents (whole-vacuole configuration) and single-channel currents (cytosolic side-out configuration) were recorded in a symmetrical 100 mM KCl and Ca^2+^ gradient (0.1 mM in the bath and 0 mM in the pipette). It should be added that we decided to use symmetrical 100 mM KCl for two reasons – firstly, because at symmetrical 100 mM KCl, which is very often used in patch-clamp experiments, the K^+^ current flows from the cytosol to the vacuole and secondly because at this concentration of KCl, auxin, immediately (3 min) after its addition, causes a decrease in the volume of the vacuoles. The macroscopic current recordings showed slow activation (Fig. 2a, control) and strong outward rectification of the steady-state currents at voltages that were more positive than + 20 mV (Fig. 2b, control). When IAA at a final concentration of 1 μM was added to the bath solution, the SV currents increased at all potentials between + 20 and + 100 mV compared to the control at 5 min (Fig. 2b). For example, in the whole-vacuole configuration, the addition of 1 μM IAA resulted in a 60% increase in the current amplitudes at 100 mV compared to the control at 5 min (I_norm_ = 0.49 ± 0.07 SE for control 5 min and I_norm_ = 0.79 ± 0.1 SE for 1 μM IAA 5 min). Interestingly, at concentrations 0.1 and 10 μM IAA cause only slight changes of steady-state current as compared to the control at 5 min. Therefore for further electrophysiological experiments 1 μM concentration of IAA was chosen. As is presented in Fig. 2, the activity of ion channels can be lost during patch-clamp experiments, which is known as “run down”. The “run down” defined as inhibition of SV channel activity in time, particularly visible in the whole-vacuole configuration, is a common phenomenon described in numerous publications [20–27]. However the opposite effect i.e. the increase of SV channel activity in time was also observed [28]. Fig. 3 presents the time constants, τ, of the monoexponential function fitted to the time courses of the macroscopic SV currents that were recorded in the presence and absence of IAA. These constants can be interpreted as the rate of the SV channel activation after the application of a voltage pulse. Fig. 3 indicates that at voltages between + 60 and + 90 mV, the time constants, τ, decrease in the presence of IAA by ca. 30% (for example at + 80 mV, τ = 1.146 ± 0.067 SE for control 5 min and τ = 0.884 ± 0.09 SE for IAA 5 min, at + 70 mV, τ = 1.298 ± 0.077 SE for control 5 min and τ = 0.851 ± 0.066 SE for IAA 5 min,), thus suggesting faster channel activation with IAA. At 40, 50 and 100 mV the time constant did not depend on IAA. When considering the microscopic currents in the cytosolic side-out configuration (Fig. 4), channels that had a higher current amplitude could be recorded in the presence of IAA compared to the control at 5 min. This is evident in Fig. 5, which shows that at voltages between 80 and 100 mV auxin significantly increased the amplitude of the SV currents compared to the control at 5 min (for example at + 100 mV *I* = 1.957 ± 0.388 SE for control 5 min and *I* = 2.762 ± 0.124 SE for IAA 5 min). Taking into account the density of the SV channels calculated as whole-vacuole current divided by current of the single channel and surface area of the vacuole, IAA increased number of active SV channels as compared to the control after 5 min. For example, the density of the channels in control at 0 min amounted 795 channels per 1000 μm^2^ while 5 min later this parameter was 600 channels per 1000 μm^2^. In the presence of IAA 5 min after its addition the density of the channels was equal 724 per 1000 μm^2^ (the density values were calculated for + 100 mV). All of the points in the scatter plots (Fig. 6), which show the distribution of the times of the different current state events as a function of the amplitude of the current, indicate the events of the closing or opening of one, two, three and four SV channels. As can be seen at Fig. 6a the current amplitude of single channels in the presence of IAA is maintained at the level comparable with that recorded for the control at 0 min. Fig. 6b and c, which show the average values of the times and the number of events (closed, open) versus the current level, respectively do not show the significant difference between control and IAA after 5 min. As can be seen in Fig. 7, the open probability of single channels at 80 mV was threefold higher in the presence of IAA compared to the control at 5 min, while at the remaining voltages, it was similar to the control values.

Taken together, our electrophysiological data suggest that in a whole-vacuolar configuration, (1) auxin at 1 μM enhanced the SV channel activity compared to the control, (2) the time constant, τ, in the range 40–90 mV decreased in the presence of IAA at 1 μM, (3) auxin at 1 μM increased the amplitude of the SV currents compared to the control and (4) the open probability of single channels was only significantly higher at 80 mV compared to the control.

## Discussion

The tonoplast regulates the traffic of ions and metabolites between the cytosol and the vacuole, which are necessary for plant cell growth. In recent few years the interest in vacuoles increased also in the aspect of auxin action in plant cell growth and development. The identification of tonoplast permease WAT1 transporting auxin out of the vacuole and the inverse correlation between auxin content in plant cell and its vacuolation status give new insight on role of vacuoles in auxin homeostasis [6, 29]. As hitherto interest focused on the role of vacuoles in auxin redistribution in the cell, our goal was to determine the auxin impact on vacuole volume changes and probable contribution of tonoplast cation channels (TPC1/SV) in this process.

In our experiments, the vacuoles that had been incubated in a medium without K^+^ and 1 μM IAA were found to swell (about 8%) over first 60 min, while a significantly faster increase in the volume of the vacuoles (ca. twofold) was observed in the presence of 1 μM IAA (Fig. 1). The fact that the increase or decrease of the volume of the vacuoles is sensitive to IAA and depends on the K^+^ concentration suggests that the electrical potential of the tonoplast may play a role in this phenomenon. It was previously shown that at high external potassium concentrations, which are comparable to cytosolic values, the membrane potential of the vacuoles that had been isolated from the storage roots of red beet was almost completely abolished, whereas at 0 mM K^+^, it was around + 75 mV [30, 31]. The electrical potential and the pH gradient across the tonoplast provide the driving forces for the transport and accumulation of metabolites and ions in the vacuolar lumen. However, to date, it is not clear how auxin changes the ion transport across the vacuolar membrane of plant cells. Taking the above into account, we performed experiments in which the effect of auxin (IAA) on the SV channel activity in the vacuolar membrane of red beet vacuoles was studied. It is well established that these channels and H^+^ pumps represent the major conductance of the vacuolar membrane (reviewed in [13, 32]). Here, we demonstrate that under the experimental conditions of this study (symmetrical 100 mM KCl and Ca^2+^ gradient), vacuoles that had been isolated from the red beet taproot were characterized by SV channels whose electrical properties, such as slow activation and outward rectification, are close to those that were previously described in *Beta vulgaris* taproots [14, 33, 34]. The addition of 1 μM IAA to the bath solution enhanced the SV currents compared to the control (Fig. 2). It is suggested that the stimulation of the macroscopic SV currents that were observed in the presence of IAA may indicate that auxin either acts directly as a channel activator or that it indirectly alters the kinetics of the transition between the closed and open state of a channel. Analysis of the kinetics of the relaxation of macroscopic current may be a source of information on channel gating. Comparing the data that was obtained from the whole-vacuole and single channel recordings, it might be suggested that IAA at 1 μM enhanced the SV currents as a result of the faster channel activation, the increased amplitude of SV currents and a higher open probability of single channels at 80 mV.

In order to explain the mechanism of the modulation of the SV channel by auxin, two scenarios are possible: (1) at the pH of the incubation medium used in the experiments (pH 7.5), the anionic form of IAA (IAA^−^) predominates, which can interact with the voltage sensing S10 domain, thus causing changes in the gating kinetics of the SV channel (for the structure of the TPC1 channel, see [17–19] and (2) because auxins are able to interact with lipids and change the properties of the lipid bilayer [35–37], they may disturb the interaction between lipids and proteins and therefore indirectly modulate the activity of the SV channels.

When comparing our electrophysiological experiments with ones in which the diameters of vacuoles were measured, it should be concluded that IAA at 1 μM increased the SV channel currents while it decreased the volume of the vacuoles. Taking the above into account, it might be hypothesized that in the presence of IAA, the SV channels play a role in the intracellular space-filling function of the vacuole (“vacuolar balloon”). In agreement with our hypothesis, the IAA-induced uptake of K^+^ [2] and probably Cl^−^ ([38] and references therein) into the cytoplasm of plant cells might be partly (apart from maintenance of turgor pressure of expanding cell) compensated for by a decrease in the vacuole volume in order to maintain cytoplasm homeostasis. Nevertheless the results presented in this paper indicate that there is no simple interrelation between SV channels activity and volume changes of the vacuoles in the presence of IAA.

## Conclusions

Taken together, our results suggest that auxin enhances the SV currents in red beet vacuoles as a result of a faster channel activation, an increased amplitude of SV currents and a higher open probability of single channels at 80 mV, thus simultaneously causing a decrease in vacuole volume. It is suggested that auxin (IAA)), at least at 1 μM, modulate the SV channels and the volume of red beet taproot vacuoles.

## Methods

### Plant material and vacuole isolation

Red beet (*Beta vulgaris* L.) taproots vacuoles were isolated using the nonenzymatic method that was previously described by Coyaud et al. [39]. In the experiments in which the diameter of the vacuoles or SV channel activity were measured, the vacuoles were mechanically isolated directly onto glass slides or into an electrophysiological chamber (1 ml in volume) by rinsing the surface of fresh tissue slices with the bath solution.

### Vacuole volume measurements

Vacuole diameters were measured using an Ax70 microscope (Olympus Provis) with a fully automatic photomicrography that was connected to a camera (Hammamatsu, Japan). The volume of the vacuoles was measured in a bath solution containing: (1) 100 mM K^+^ (100 mM KCl, 2 mM MgCl_2_, 0.1 mM CaCl_2_, 2 mM DTT, 5 mM MES, 5 mM Tris and 400 mM sorbitol, pH 7.5, osmolality 650 mOsm), (2) 20 mM K^+^ (20 mM KCl, 2 mM MgCl_2_, 0.1 mM CaCl_2_, 2 mM DTT, 5 mM MES, 5 mM Tris and 460 mM sorbitol, pH 7.5, osmolality 650 mOsm) and (3) 0 mM K^+^ (0 mM KCl, 2 mM MgCl_2_, 0.1 mM CaCl_2_, 2 mM DTT, 5 mM MES, 5 mM Tris and 600 mM sorbitol, pH 7.5, osmolality 650 mOsm).

### Patch-clamp measurements

The electrophysiological experiments were performed in whole-vacuole and excised cytosolic side-out patch configuration. The recordings were made using an EPC-7 Plus amplifier (List-Medical-Electronic, Darmstadt, Germany), as was recently described by Trela et al. [40]. For signal filtration a five-pole Bessel filter was used with sampling frequency of 1 to 100 kHz. The patch pipettes were prepared from borosilicate glass tubes (Kimax-51, Kimble Products, Toledo, Ohio, USA) in accordance with the procedure previously described by us [40]. Voltage pulse within a range of 300 to 900 mV combined with gentle suction let gain the access to the vacuole interior. The current and voltage signs were in accordance with the convention proposed by Bertl et al. [41].

The control bath solution in the patch-clamp experiments was the same as the one that was used for the vacuole diameter measurements (solution number 1). The pipettes were filled with a solution containing 100 mM KCl, 2 mM MgCl_2_, 5 mM MES, 5 mM Tris and pH 5.5, which was adjusted to an osmolality of 580 mOsm with sorbitol. The osmolality of all of the media used during the measurements was adjusted by a cryoscopic osmometer (Semi-Micro Osmometer K-7400, Knauer, Germany). It is well established that under symmetrical 100 mM K^+^ and micromolar cytosolic Ca^2+^ concentrations, the SV channels K^+^ current is outwardly directed. Nevertheless at a calcium gradient (0.1 mM in the bath and 0 mM in the pipette), a Ca^2+^current into the vacuole cannot be excluded at depolarizing voltages. Due to the lack of specific inhibitors of these channels (for a review see [13]) separation of the K^+^ and Ca^2+^ currents through the SV channels is difficult. The effect of indole-3-acetic acid (IAA) on the SV channels and the volume of the vacuoles were studied. Concentration of IAA (1 μM) was selected from among three tested concentrations (0.1, 1 and 10 μM) as the one that caused the most significant increase in vacuole volume and change in SV activity in whole-vacuole configuration. In the experiments, the control bath was changed for a new one with the same salt composition that additionally contained IAA at a final concentration of 1 μM. The exchange of the bath solution in the recording chamber was accomplished through the continuous perfusion of the measuring chamber using an SP200 infusion pump (World Precision Instruments, USA). All of the experiments were carried out at room temperature (22 ± 1 °C).

Data were stored and elaborated using PatchMaster, FitMaster (HEKA Electronic, Lambrecht, Germany) and Dell Statistica (data analysis software system), version 13. Analysis of the results was performed in accordance with our earlier paper [40]. The opening probability was calculated as the total opening time normalized to the total recording time and the number of active channels in a patch [42].

## Abbreviations

AtTPC1: *Arabidopsis thaliana* two-pore channel 1
IAA: Indole-3-acetic acid
Na_v_Ab: voltage-gated sodium from *Arcobacter butzleri*
PM: Plasma membrane
SV: Slow vacuolar channels
TPC1: Two-pore channel 1
WAT1: Walls are thin1
ZMK1: *Zea mays* K^+^ channel 1
